# Binding of the protein ICln to α-integrin contributes to the activation of ICl_swell_ current

**DOI:** 10.1038/s41598-019-48496-4

**Published:** 2019-08-21

**Authors:** Andreas Schedlbauer, Grazia Tamma, Simona Rodighiero, Davide Antonio Civello, Margherita Tamplenizza, Karin Ledolter, Charity Nofziger, Wolfgang Patsch, Robert Konrat, Markus Paulmichl, Silvia Dossena

**Affiliations:** 1 Department of Structural and Computational Biology, Max F. Perutz Laboratories, University of Vienna, 1030 Vienna, Austria; 2 Department of Biosciences, Biotechnologies and Biopharmaceutics, University of Bari Aldo Moro, 70126 Bari, Italy; 3 Istituto Nazionale di Biostrutture e Biosistemi, 00136 Rome, Italy; 4 Department of Experimental Oncology, European Institute of Oncology, 20141 Milan, Italy; 5 Institute of Pharmacology and Toxicology, Paracelsus Medical University, 5020 Salzburg, Austria; 6 Tensive S.r.l., 20139 Milan, Italy; 7 PharmGenetix Gmbh, 5081 Niederalm, Austria; 8 Department of Personalized Medicine, Humanomed, 9020 Klagenfurt, Austria; 9 Present Address: Centro de Investigación Cooperativa en Biociencias (CIC bioGUNE), Bizkaia Science and Technology Park, 48160 Derio (Bizkaia), Spain

**Keywords:** Protein-protein interaction networks, Patch clamp

## Abstract

ICl_swell_ is the chloride current induced by cell swelling, and plays a fundamental role in several biological processes, including the regulatory volume decrease (RVD). ICln is a highly conserved, ubiquitously expressed and multifunctional protein involved in the activation of ICl_swell_. In platelets, ICln binds to the intracellular domain of the integrin αIIb chain, however, whether the ICln/integrin interaction plays a role in RVD is not known. Here we show that a direct molecular interaction between ICln and the integrin α-chain is not restricted to platelets and involves highly conserved amino acid motifs. Integrin α recruits ICln to the plasma membrane, thereby facilitating the activation of ICl_swell_ during hypotonicity. Perturbation of the ICln/integrin interaction prevents the transposition of ICln towards the cell surface and, in parallel, impedes the activation of ICl_swell_. We suggest that the ICln/integrin interaction interface may represent a new molecular target enabling specific ICl_swell_ suppression in pathological conditions when this current is deregulated or plays a detrimental role.

## Introduction

Cellular volume changes occur in a variety of physiological processes, including transepithelial transport, cell migration, proliferation, and death. As limiting excessive or prolonged volume changes is essential for normal cell function and survival, most cell types are able to counteract volume perturbations by initiating the homeostatic processes of regulatory volume increase and decrease (RVI and RVD, respectively)^1–4^. Activation of a chloride conductance upon cell swelling (ICl_swell_) is a key step in RVD and occurs *via* volume regulated anion channels (VRACs)^5,6^.

The mechanisms of osmosensing are complex and far from being completely understood. Several lines of evidence implicate integrins as upstream sensors of cell volume perturbations in mammalian cells after cell swelling or shrinkage^1,7–9^. Integrins are a highly conserved family of heterodimeric adhesion molecules that consist of an α- and a β-subunit and connect the extracellular matrix to intracellular signalling proteins and the cytoskeleton, and play a critical role in many vital cellular functions, such as cell adhesion, migration, invasion, differentiation, proliferation, apoptosis, growth-factor signalling and response to hypotonicity^10–13^. By linking the exterior and interior space of cells, integrins permit a bidirectional transmission (outside-in and inside-out signalling) of mechanical and biochemical stimuli across the plasma membrane^14,15^. Specifically, activation of integrins by hypotonicity^16^ seems to trigger Cl^−^ and organic osmolyte fluxes during RVD, possibly by recruitment of a member of the SRC family of kinases^17^. Integrins also establish an intricate network of molecular interactions *via* their intracellular domains. The β-subunits are major players in this context, and bind signalling hubs such as talin and kindlins^18–20^. The intracellular network of the α-subunits was less extensively studied. Evidence was gathered that the intracellular domain of the platelet-specific integrin αIIb physically interacts with the protein ICln. This interaction is part of the molecular events underlying platelet activation and capacity to form a thrombus^21,22^.

ICln^23^ is a highly conserved, ubiquitously expressed and multifunctional protein that plays a critical role in cell homeostasis and survival^24^. ICln was suggested to be the channel or part of the macromolecular complex underlying the activation of ICl_swell_^25,26^. Despite being mainly located in the cytosol in resting cells, ICln is transposed towards the plasma membrane upon hypotonic challenge^27,28^ or other stimuli^29^. In addition, ICln establishes complex interactions with diverse proteins belonging to different subcellular compartments and functional modules^21,28,30–32^. For instance, ICln regulates the process of splicing and small nuclear ribonucleoproteins (snRNP) biogenesis by associating with several Smith antigen (Sm) proteins of the spliceosomal core^33^ and controlling their methylation^34–37^.

The cytoplasmic tail of human integrin αIIb contains the highly conserved motif (1020)KVGFFKR(1026) (single letter amino acid code; the numbers indicate the amino acid positions within the primary sequence of human αIIb integrin; NCBI Reference Sequence: NP_000410.2), which regulates integrin activation and acts as a recognition site for various intracellular proteins, including ICln^22^. As the KxGFFKR motif (x denotes any amino acid) is common to several α integrin isoforms^38^, it is important to understand whether the ICln/integrin interaction can also occur in nucleated cells and identify the corresponding function. Here we show that the interaction between ICln and the intracellular domain of integrin α-chain is essential for the activation of ICl_swell_, and is independent from the occupancy of integrin α by extracellular ligands.

## Results

### ICln establishes a direct molecular interaction with the intracellular domain of the integrin αIIb chain

**Figure 1:**
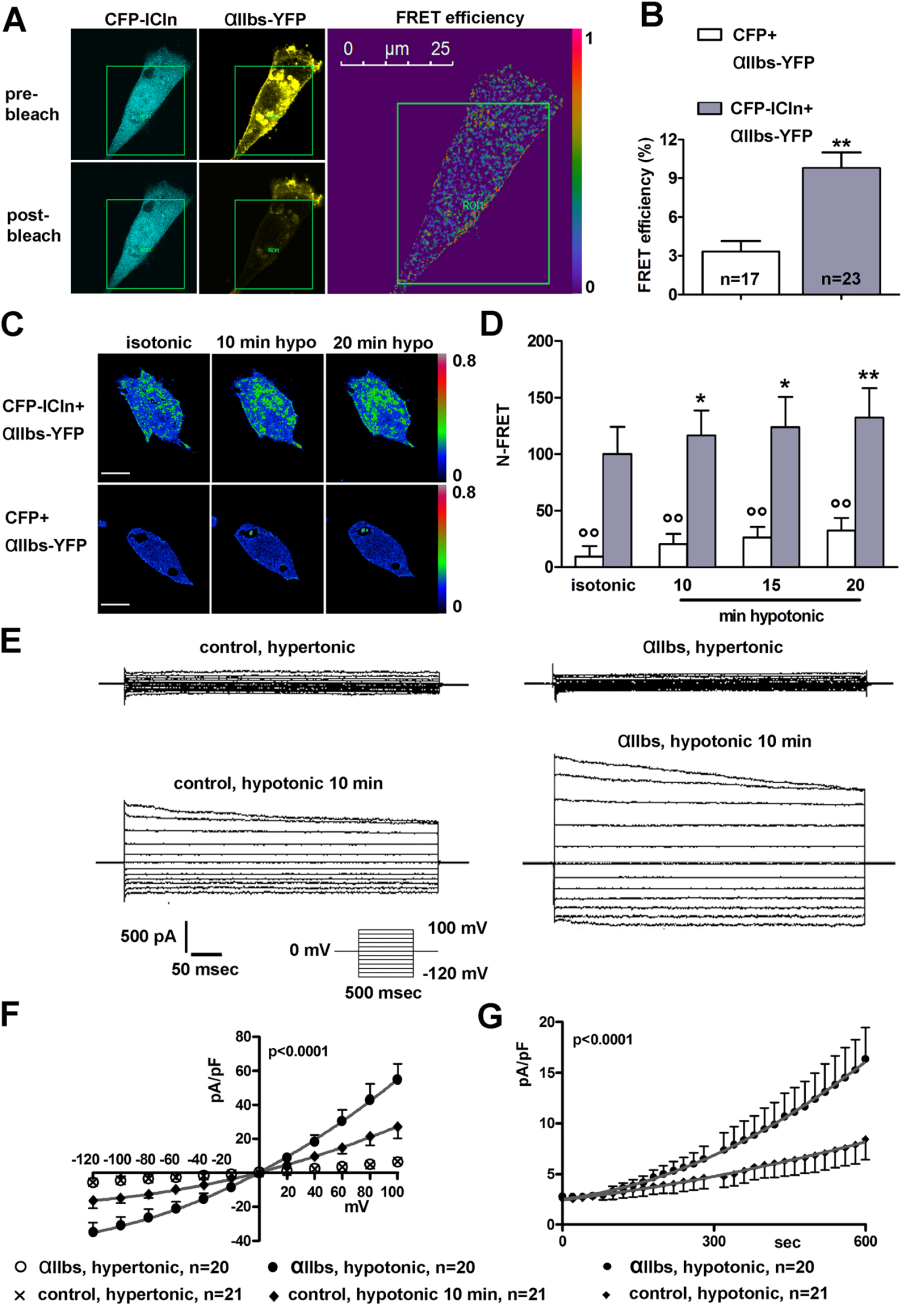
The integrin α-chain interacts with ICln and facilitates the activation of ICl_swell_. Living NIH-3T3 fibroblasts were transiently transfected with *Canis familiaris* ICln and αIIb_s_ and bathed in isotonic solution. (**A**) The FRET donor (CFP) and the FRET acceptor (YFP) were imaged before and after photobleaching of the acceptor. The green squares correspond to the bleaching ROI and the right panel represents the FRET efficiency image. (**B**) FRET efficiency of CFP-ICln and αIIb_s_-YFP or CFP and αIIb_s_-YFP determined in three different plasma membrane regions of interest (ROIs) for each cell. n represents the number of cells. **p < 0.01, unpaired Student’s t test. (**C**) FRET signal of CFP-ICln and αIIb_s_-YFP or CFP and αIIb_s_-YFP determined by sensitized emission in living NIH-3T3 fibroblasts kept in isotonic or hypotonic solution for 10 and 20 min. Bar: 10 µm. (**D**) N-FRET of CFP-ICln and αIIb_s_-YFP (n = 8) or CFP and αIIb_s_-YFP (n = 6) in isotonic solution or after 10, 15 and 20 min of hypotonic stress. Data are normalized for the value of N-FRET of CFP-ICln and αIIb_s_-YFP measured in isotonic solution and expressed in %. Hypotonic versus isotonic: *p < 0.05, **p < 0.01, paired Student t test; CFP-ICln versus CFP: °°p < 0.01, unpaired Student t test. n indicates the number of cells. Caption in **B** also refers to **D**. (**E**) Original recordings obtained by whole-cell patch-clamp in hypertonic (top) or hypotonic (bottom) conditions in control (left panels) or αIIb_s_ -transfected (right panels) HEK293 Phoenix cells stimulated with voltage increments as depicted in the lower inset. Cells over-expressed both αIIb_s_ and the transfection marker EGFP as separate proteins, or EGFP only. (**F**) The current density-to-voltage relationships measured 10 min following hypotonic shock, and **G**, the current density-to-time relationships were fitted with second order polynomials, following application of the extra-sum of squares F test (αIIb_s_, hypotonic, versus control, hypotonic: p < 0.0001).

To verify whether the ICln-integrin interaction is restricted to the process of platelet activation, we performed fluorescence resonance energy transfer (FRET) experiments in living NIH-3T3 fibroblasts kept in isotonic solution and transiently transfected with *Canis familiaris* ICln and the transmembrane and intracellular portion (amino acids 987–1039) of the human integrin αIIb chain (αIIb-short, αIIb_s_) (Fig. 1A,B). Deletion of the extracellular portion of αIIb avoided perturbation of a possible ICln/αIIb interaction by an extracellular signal. The cDNA sequences coding for ICln and αIIb_s_ were cloned into vectors to produce fusion proteins with fluorophores suitable for FRET experiments, *i.e*. CFP-ICln and αIIb_s_-YFP respectively (see Methods). After expression, αIIb_s_-YFP clearly targeted to the plasma membrane, with a partial retention in intracellular compartments (Fig. 1A and Supplementary Fig. S1). The FRET efficiency of CFP-ICln and αIIb_s_-YFP determined in the plasma membrane region of transfected cells following photobleaching of the FRET acceptor YFP was significantly higher (p < 0.01) with respect to that of the corresponding negative control (expression of CFP alone and αIIb_s_-YFP) (Fig. 1B, Supplementary Table S1 and Fig. S1). Similar experiments performed on NIH-3T3 fibroblasts fixed in 3% paraformaldehyde and kept in a phosphate buffered saline led to similar results (data not shown). Also in HEK293 Phoenix cells, the FRET efficiency of CFP-ICln and αIIb_s_-YFP determined in the plasma membrane region was significantly higher than that determined in control cells transfected with CFP and αIIb_s_-YFP (Supplementary Table S1). The ratio of the FRET efficiency between CFP-ICln and αIIb_s_-YFP and the stochastic interaction given by the FRET efficiency between CFP and αIIb_s_-YFP in the two cells types was similar, being 2.9 and 2.7 in NIH-3T3 and HEK293 Phoenix cells, respectively. Accordingly, in sensitized emission N-FRET experiments, ICln and αIIb_s_ produced an N-FRET in isotonic solution that was significantly higher (p < 0.01) compared to the control (Fig. 1C,D, isotonic, and Supplementary File S1). Following exposure of cells to a hypotonic bath solution, the N-FRET of ICln and αIIb_s_ significantly increased over time, while FRET of CFP and αIIb_s_-YFP did not significantly change (Fig. 1C,D and Supplementary File S1). These results show that in isotonic, as well as hypotonic conditions, ICln is located in close proximity to αIIb_s_, and therefore strongly suggest a direct molecular interaction between the two molecules.

### Over-expression of αIIb_s_ activates ICl_swell_

To test whether the interaction between ICln and the integrin α chain might have a functional implication on ICl_swell_, the whole-cell patch-clamp technique was used. HEK293 Phoenix cells were transfected with the pIRES2-EGFP vector with or without αIIb_s_ cDNA (see Methods). The bicistronic αIIb_s_-pIRES2-EGFP vector was used to obtain the simultaneous expression of αIIb_s_ and the transfection marker EGFP as two independent proteins in the same cell, thus allowing for the identification of cells expressing αIIb_s_ by fluorescence microscopy. After the seal was realized and the whole-cell configuration was obtained, ICl_swell_ was elicited by reducing the extracellular osmolarity. Hypotonic shock induced the activation of a large chloride current with the biophysical fingerprints of ICl_swell_, characterized by outward rectification and slow voltage- and time-dependent inactivation at positive potentials^6^ in both control and αIIb_s_ over-expressing cells (Fig. 1E). The current density-to-voltage relation determined after a 10 min exposure to extracellular hypotonic solution (Fig. 1F), as well as the current density-to-time relation (Fig. 1G), showed a strong up-regulation of ICl_swell_ in cells over-expressing αIIb_s_ compared to control cells (p < 0.0001). These results suggest an involvement of the αIIb chain in the activation of ICl_swell_.

### The cell-permeable (1020)KVGFFKR(1026) integrin peptide impairs the translocation of ICln to the plasma membrane and the activation of ICl_swell_

**Figure 2:**
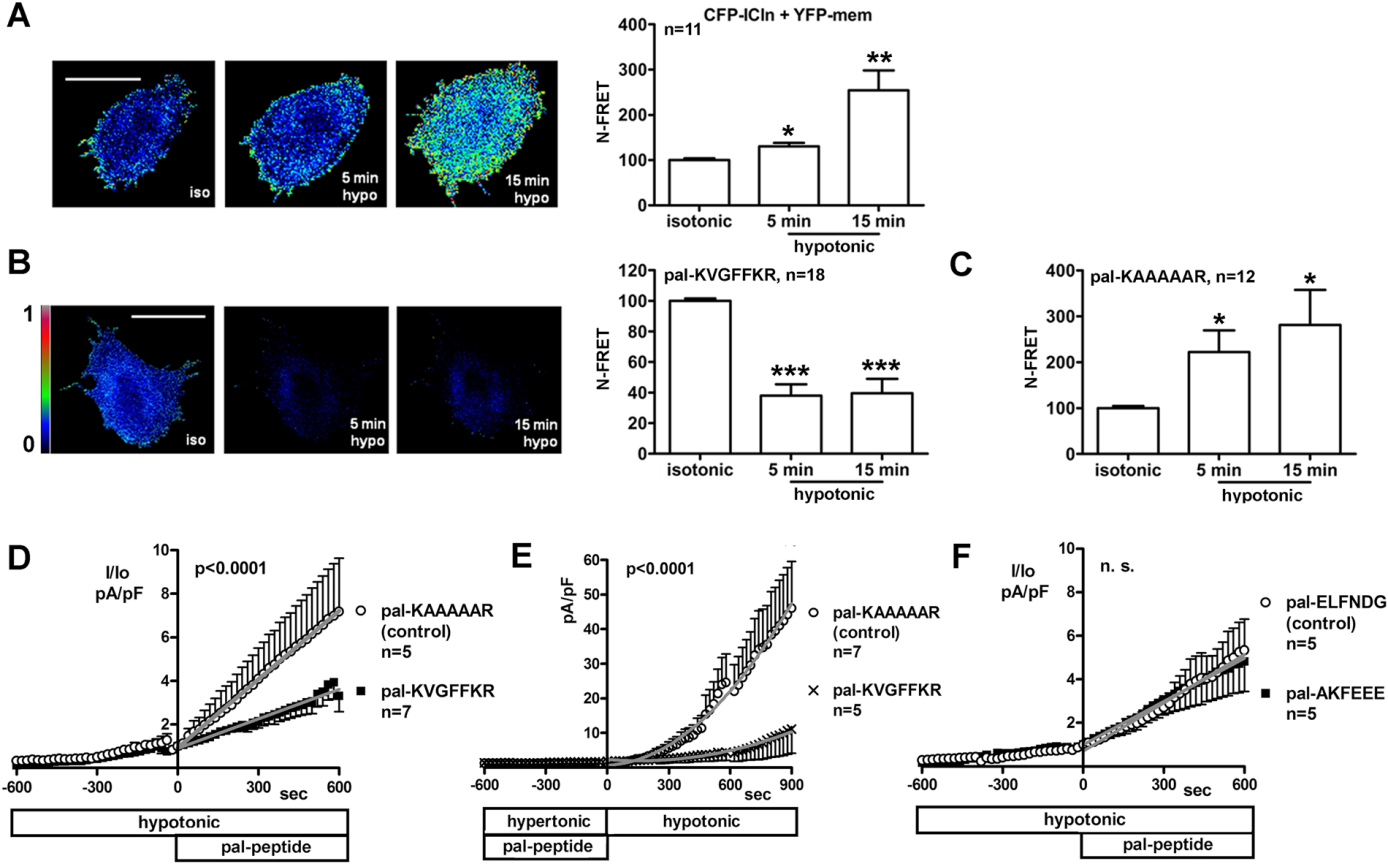
The palmitoylated (1020)KVGFFKR(1026) integrin peptide impairs the translocation of ICln and the activation of ICl_swell_. Living NIH-3T3 fibroblasts were transiently transfected with CFP fused to the Nt of human ICln (CFP-ICln) and the membrane label YFP-mem. Cells were imaged in isotonic solution and after 5 and 15 min of hypotonic stress. FRET signal (left panels) and N-FRET (right graphs) of CFP-ICln and YFP-mem determined by sensitized emission in cells (**A**) left untreated or (**B**) pre-incubated for 10 min in isotonic solution with 100 µM of the synthetic palmitoylated (1020)KVGFFKR(1026) peptide or (**C**) the synthetic palmitoylated KAAAAAR peptide. Data were normalized for the FRET value measured in isotonic solution and expressed in %. *p < 0.05, **p < 0.01, ***p < 0.001, paired Student’s t-test. The bar in the left panels corresponds to 10 µm. The pseudo-colours LUT shown in B also applies to A. n indicates the number of cells assayed. (**D–F**) Current density-to-time relationships of ICl_swell_ in HEK293 Phoenix cells measured by patch-clamp in whole-cell configuration following a hypotonic stimulus. In (**D,F**) 100 µM of the indicated palmitoylated peptides were added to the bath after partial activation of ICl_swell_ by a 10 min exposure of cells to the hypotonic solution. In (**E**) cells were pre-incubated for 10 min in a hypertonic solution containing 100 µM of the palmitoylated peptides, and then stimulated with hypotonic solution. In (**D,F**) the current density (I) was normalized for the current value measured immediately before the addition of the peptides to the bath (I_0_). Data were fitted by linear regression (**D,F**) or with second order polynomials (**E**), following application of the extra-sum of squares F test (in **D** and **E**, KVGFFKR versus KAAAAAR: p < 0.0001; in **F**, AKFEEE versus ELFNDG: not significant).

The translocation of ICln to the plasma membrane after hypotonic challenge was verified by sensitized emission N-FRET in living NIH-3T3 fibroblasts exposed to the synthetic palmitoylated peptide (1020)KVGFFKR(1026), corresponding to the amino acid sequence of the ICln recognition domain within the integrin αIIb chain^22^. Control cells were treated with the synthetic palmitoylated peptide KAAAAAR. Palmitoylation allows the peptides applied to the extracellular solution to access the intracellular environment^22,39^, with preferential, although not exclusive, targeting to the plasma membrane. Cells expressed the fusion protein CFP-ICln and the membrane label YFP-mem (*i.e*. the YFP protein tagged with an N-terminal palmitoylation sequence, see Methods). As previously reported^27,28^, hypotonicity induced a significant increase of FRET between CFP-ICln and YFP-mem, thereby indicating a transposition of ICln towards the cell membrane (Fig. 2A). Interestingly, pre-incubation of cells with the palmitoylated (1020)KVGFFKR(1026) peptide not only prevented the expected FRET increase following hypotonic stress, but indeed led to a FRET decrease. This indicates that the peptide not only abolished the translocation of ICln normally observed in hypotonicity, but additionally decreased the amount of the membrane-bound ICln compared to isotonic conditions (Fig. 2B). In contrast, preincubation of cells with the palmitoylated KAAAAAR control peptide did not affect the hypotonicity-induced translocation of ICln towards the plasma membrane (Fig. 2C). Importantly, patch-clamp experiments in whole-cell configuration showed that the impaired translocation of ICln toward the cell surface was paralleled by an impaired activation of ICl_swell_. Specifically, the palmitoylated (1020)KVGFFKR(1026) peptide applied to the bath solution, and not the palmitoylated KAAAAAR peptide, dramatically impaired (Fig. 2D) or prevented (Fig. 2E) the activation of ICl_swell_ in hypotonicity-stimulated HEK293 Phoenix cells. Larkin *et al*. previously speculated that the amino acids (84)AKFEEE(89) of ICln might be involved in binding the (1020)KVGFFKR(1026) motif^22^. However, the cell-permeable peptide (84)AKFEEE(89) did not significantly affect ICl_swell_ activation compared to the unrelated cell-permeable peptide ELFNDG (Fig. 2F). Therefore, we concluded that the ICln amino acids (84)AKFEEE(89) unlikely represent the main integrin interacting site.

### The (61)ISLHA(65) ICln motif is the prime integrin binding site

**Figure 3:**
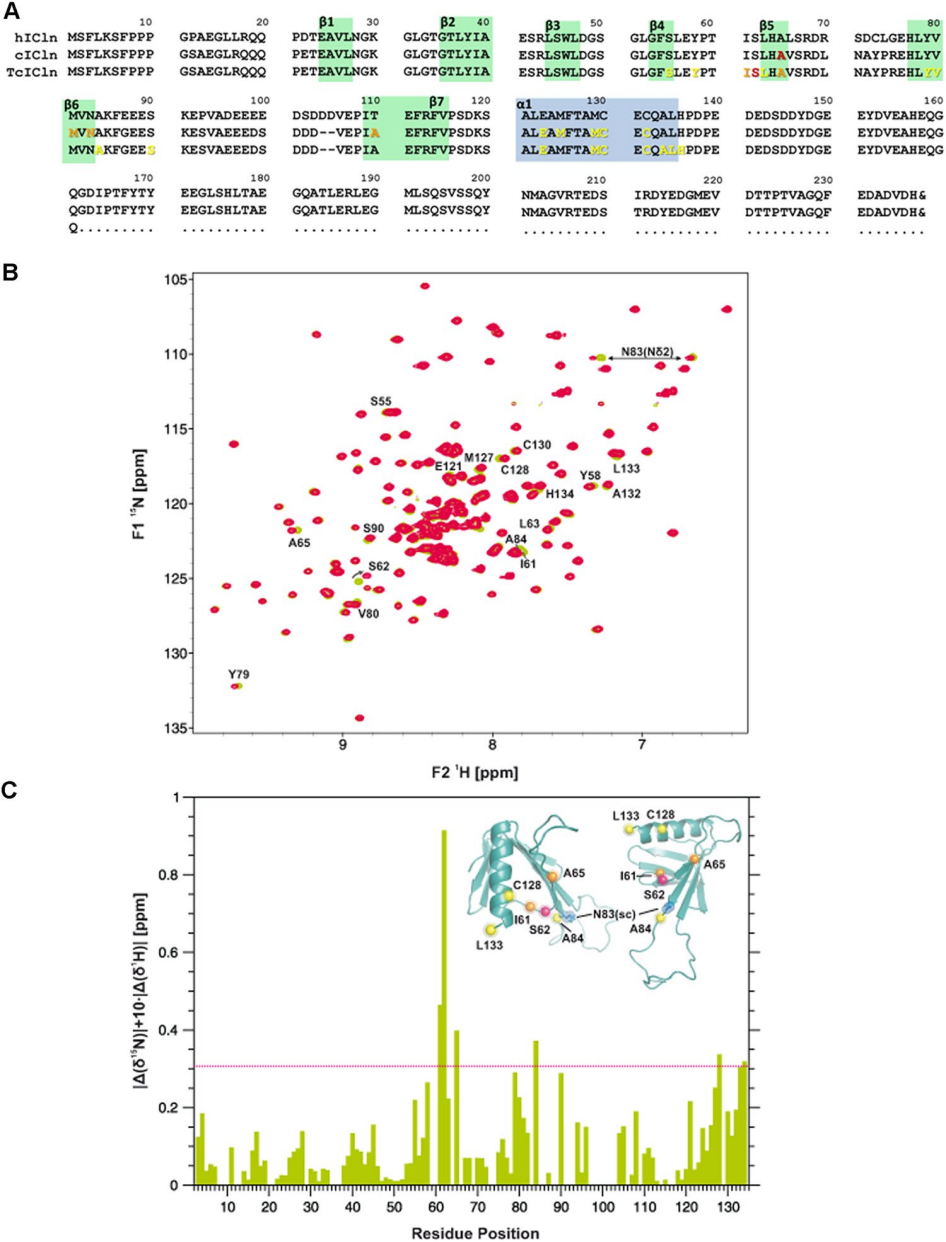
Assessment of the ICln binding site for the integrin α-chain by NMR spectroscopy. (**A**) Amino acid sequence alignment of human (h, NCBI accession NP_001284.1) and *Canis familiaris* full-length (c, NCBI accession NP_001003288.1) and truncated (Tc) ICln. The ICln amino acids showing a significant interaction with the integrin peptides are shown in color according to panel C. Green and blue boxes indicate β sheets and α helix secondary structure motifs, respectively. The amino acid numbering of TcICln and cICln after residue #103 does not correspond to the amino acid numbering of hICln due to a gap of 2 amino acids (104–105) in the cICln sequence. (**B**) Overlay of 2D ^1^H-^15^N HSQC spectra related to the apo form of TcICln (shown in green) and in the presence of an equimolar amount of the integrin peptide (1018)MWKVGFFKRNR(1028) (red). Resonances showing significant ^1^H-^15^N chemical shift changes upon ligand addition (defined applying a cutoff of |Δδ^15^N| + 10x|Δδ^1^H| > 0.2) are labelled by single letter amino acid code and their position in the primary sequence. The likewise affected signal of the side chain amine of residue N83 is indicated by a double arrow. (**C**) Distribution of the backbone amide chemical shift changes along the primary sequence and (inset) spatial position of residues of which ^1^H-^15^N resonances showed chemical shift changes 3 times higher than the standard deviation (red dashed line) represented by spheres gradiently coloured from red to yellow in the ICln model (pdb ID: 1ZYI).

In order to identify the binding site of the integrin (1020)KVGFFKR(1026) motif in ICln, we applied ^1^H-^15^N HSQC spectroscopy. Binding of the (1018)MWKVGFFKRNR(1028) integrin peptide to ICln was probed by using both full-length *Canis familiaris* ICln (cICln, Fig. 3A) and a truncated form (TcICln, Fig. 3A) lacking the C-terminal part. TcICln folds into a pleckstrin homology domain-like structure consisting of two nearly orthogonal antiparallel β-sheets forming a β-barrel capped by an α-helix near the C terminus (^40^ and Fig. 3). Figure 3B shows an overlay of NMR spectra of unligated (green) and ligated (red) TcICln. It can be seen that most of the residues are unchanged and only a subset of residues (labelled by their position in the primary sequence) display small but significant chemical shift changes. The most pronounced chemical shift changes were observed for S62 (shown in red in Fig. 3A,C), I61 and A65 (shown in orange in Fig. 3A,C). Most interestingly, a continuous stretch of residues displayed significant chemical shift changes (I61-A65). Additionally, the following residues (shown in yellow in Fig. 3A) exhibited chemical shift changes: S55, Y58, L63, Y79, V80, A84, S90 and residues in the C-terminal α-helix (E121, M127, C128, C130, A132 and L133).

Similar results were obtained for the binding of the (1020)KVGFFKR(1026) motif to full-length cICln, encompassing also the conformationally flexible C-terminus (Ct)^41^. In this experiment, the peptide (1018)MWKVGFFKRNRPPLEEDDEEGE(1039), which corresponds to the entire Ct of integrin αIIb and therefore includes the (1020)KVGFFKR(1026) motif, was used. Comparison of spectra of full-length unligated and ligated cICln shows that only a small number of peaks are shifted, while the majority of peaks remained more or less unchanged, again indicating a specific interaction. Overall, the magnitude of chemical shift changes is small, presumably due to weak binding and small structural adaptions upon ligand binding. It should also be kept in mind that ^1^H-^15^N HSQC spectra report on the protein backbone and not on side-chains, which are expected to be closer to a potential ligand. Residues which were most significantly altered upon binding were: A65, M81, N83 and A108. The most pronounced chemical shift change was observed for A65 (shown in red in Fig. 3A). It should be noted that the other residues displaying chemical shift changes (M81, N83 and A108, shown in orange in Fig. 3A) are located in the vicinity of A65 and part of a hydrogen bond network bridging the β-strands of the β-sandwich. Small chemical shift changes were observed also for residues located in the C-terminal α-helix (E121, M123, M127, C128 and C130, shown in yellow in Fig. 3A). Slight chemical shift changes found for residues in the flexible (and structurally loosely defined) loop region connecting β-strands β6 and β7 (E92 and E96) and in the flexible C-terminal tail (Y168 and T182) might be due to transient contacts of these regions with the well-folded core (PH) domain of ICln and are therefore not shown in Fig. 3A.

We thus conclude that the β4-β5 loop and the β5 β-strand comprising the ICln amino acids (61)ISLHA(65) represent the main interaction site for the (1020)KVGFFKR(1026) motif, while chemical shift changes observed for the other residues are in part due to small local structural alterations in adjacent β-strands.

### A peptide containing the (61)ISLHA(65) motif impairs the translocation of ICln to the plasma membrane and the activation of ICl_swell_

**Figure 4:**
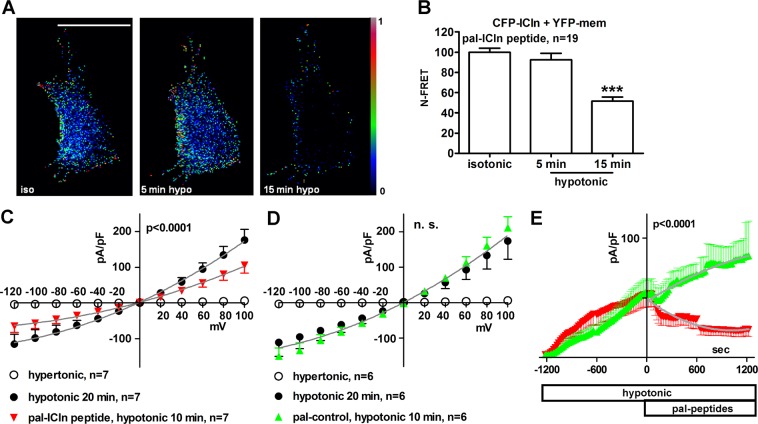
The palmitoylated (57)EYPTISLHALSRDR(70) ICln peptide impairs the translocation of ICln and the activation of ICl_swell_. Living NIH-3T3 fibroblasts were transfected with CFP fused to the Nt of human ICln (CFP-ICln) and YFP-mem. Cells were imaged in isotonic solution or after 5 and 15 min of hypotonic stress. (**A**) FRET signal and (**B**) N-FRET of CFP-ICln and YFP-mem determined by sensitized emission in cells pre-incubated for 10 min in an isotonic solution containing 50 µM of the palmitoylated (57)EYPTISLHALSRDR(70) (pal-ICln) peptide. Data were normalized for the FRET value measured in isotonic solution and expressed in %. ***p < 0.001, paired Student’s t-test. The bar in A (left panel) corresponds to 10 µm. n indicates the number of cells assayed. Current density-to-voltage relation determined by patch-clamp in whole-cell configuration in HEK293 Phoenix cells in hypertonic solution, after 20 min of hypotonic shock and 10 min after addition of 50 µM of (**C**), the palmitoylated ICln (pal-ICln) peptide or (**D**), a palmitoylated peptide with a scrambled amino acid sequence (RIYSASDLEPRLTH) as the control (pal-control) to the hypotonic bath solution. Data were fitted with second order polynomials, following application of the extra-sum of squares F test (before and after application of the ICln peptide: p < 0.0001; before and after application of the control peptide: n.s.). (**E**), current density-to-time relation of the current from experiments shown in (**C**) and (**D**). The addition of peptides to the bath was done at time zero and the current-to-voltage relationships depicted in (**C**) and (**D**) were determined at time 600 sec. The color code used in (**E**) refers to the figure caption of (**C**) and (**D**). Data were fitted with second order polynomials, following application of the extra-sum of squares F test (pal-ICln peptide versus pal-control peptide: p < 0.0001).

**Figure 5:**
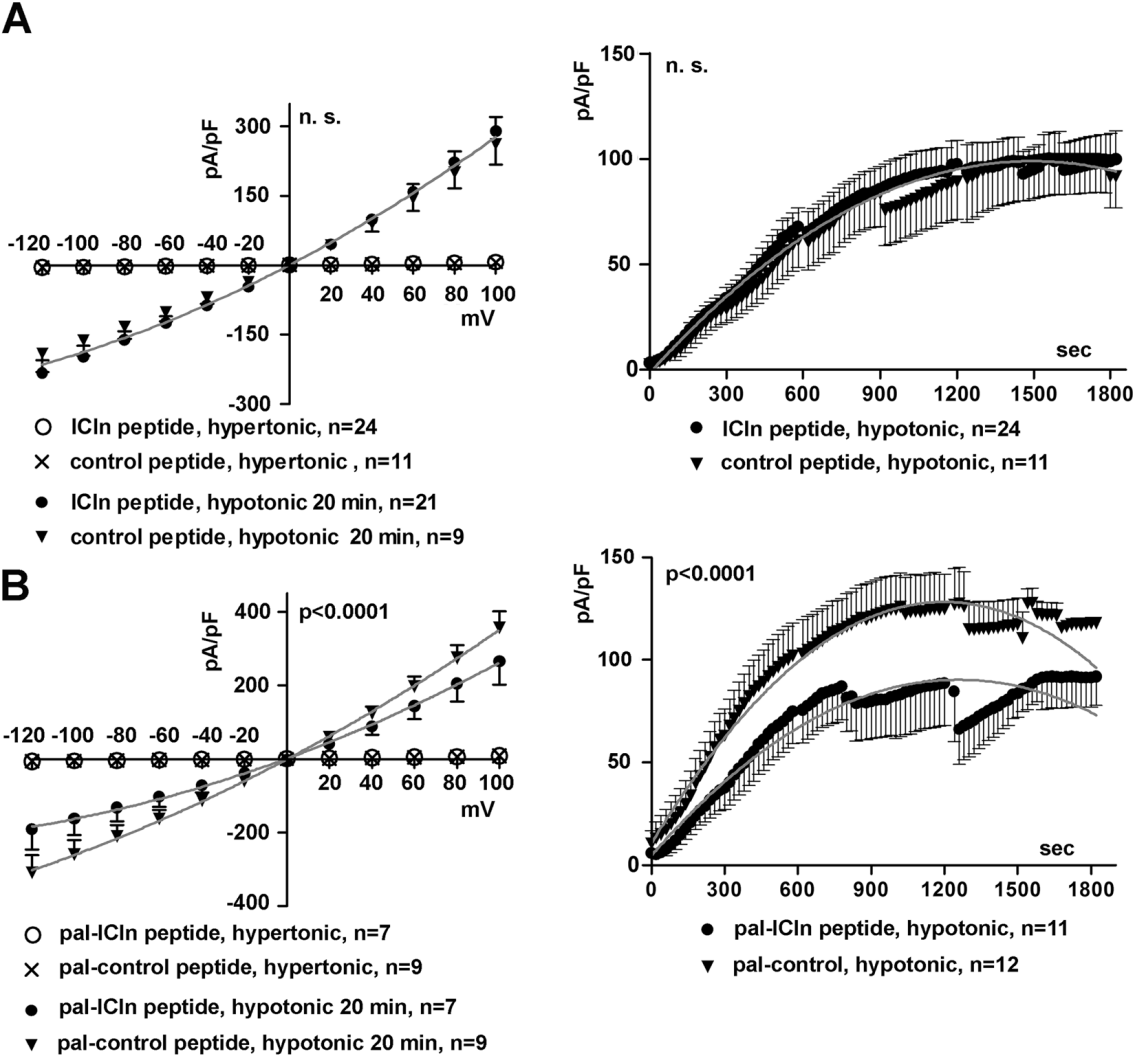
The palmitoylated (57)EYPTISLHALSRDR(70) ICln peptide applied to the intracellular solution impairs the activation of ICl_swell_. Current density-to-voltage (left) and current density-to-time (right) relationships determined by patch-clamp in whole cell configuration in HEK293 Phoenix cells in hypertonic solution and after 20 min of hypotonic shock with 50 µM of the (**A**) unmodified or (**B**) palmitoylated ICln peptides (EYPTISLHALSRDR) or control peptides (RIYSASDLEPRLTH) in the pipette filling (intracellular) solution. Data were fitted with second order polynomials, following application of the extra-sum of squares F test (hypotonic, ICln peptide *versus* control peptide: n.s.; pal-ICln peptide *versus* pal-control peptide: p < 0.0001).

We set out to verify whether a peptide comprising the ICln motif (61)ISLHA(65) could affect the transposition of ICln to the plasma membrane and the activation of ICl_swell_. The translocation of ICln to the plasma membrane after hypotonic challenge was investigated by sensitized emission N-FRET in living NIH-3T3 fibroblasts transfected with the fusion protein CFP-ICln and the membrane label YFP-mem. Pre-incubation of cells with 50 µM of the cell-permeable (palmitoylated) synthetic peptide (57)EYPTISLHALSRDR(70) (pal-ICln peptide; numbers indicate the amino acid positions within the primary sequence of hICln) encompassing the (61)ISLHA(65) motif not only abrogated the FRET increase between CFP-ICln and YFP-mem normally observed after hypotonic stress, but led to a FRET decrease compared to the isotonic condition (Fig. 4A,B), again indicating an interference with ICln transposition and localization to the plasma membrane. Accordingly, patch-clamp experiments in whole-cell configuration showed that the palmitoylated (57)EYPTISLHALSRDR(70) peptide applied to the bath (extracellular) solution (Fig. 4C,E), and not a palmitoylated peptide with a scrambled amino acid sequence (RIYSASDLEPRLTH, pal-control, Fig. 4D,E), strongly inhibited ICl_swell_ in HEK293 Phoenix cells. The effect of the peptide (57)EYPTISLHALSRDR(70) on ICl_swell_ was also investigated by adding it to the pipette filling (intracellular) solution used in patch-clamp experiments. This manoeuvre allows the peptide to gain access to the intracellular space after establishing the whole-cell configuration. The non-palmitoylated (57)EYPTISLHALSRDR(70) peptide did not interfere with the activation of the current (Fig. 5A). In contrast, adding the palmitoylated peptide to the intracellular space significantly reduced ICl_swell_ (Fig. 5B).

### Integrity of the (61)ISLHA(65) motif is essential for the association of ICln to the αIIb-chain and the activation of ICl_swell_

**Figure 6:**
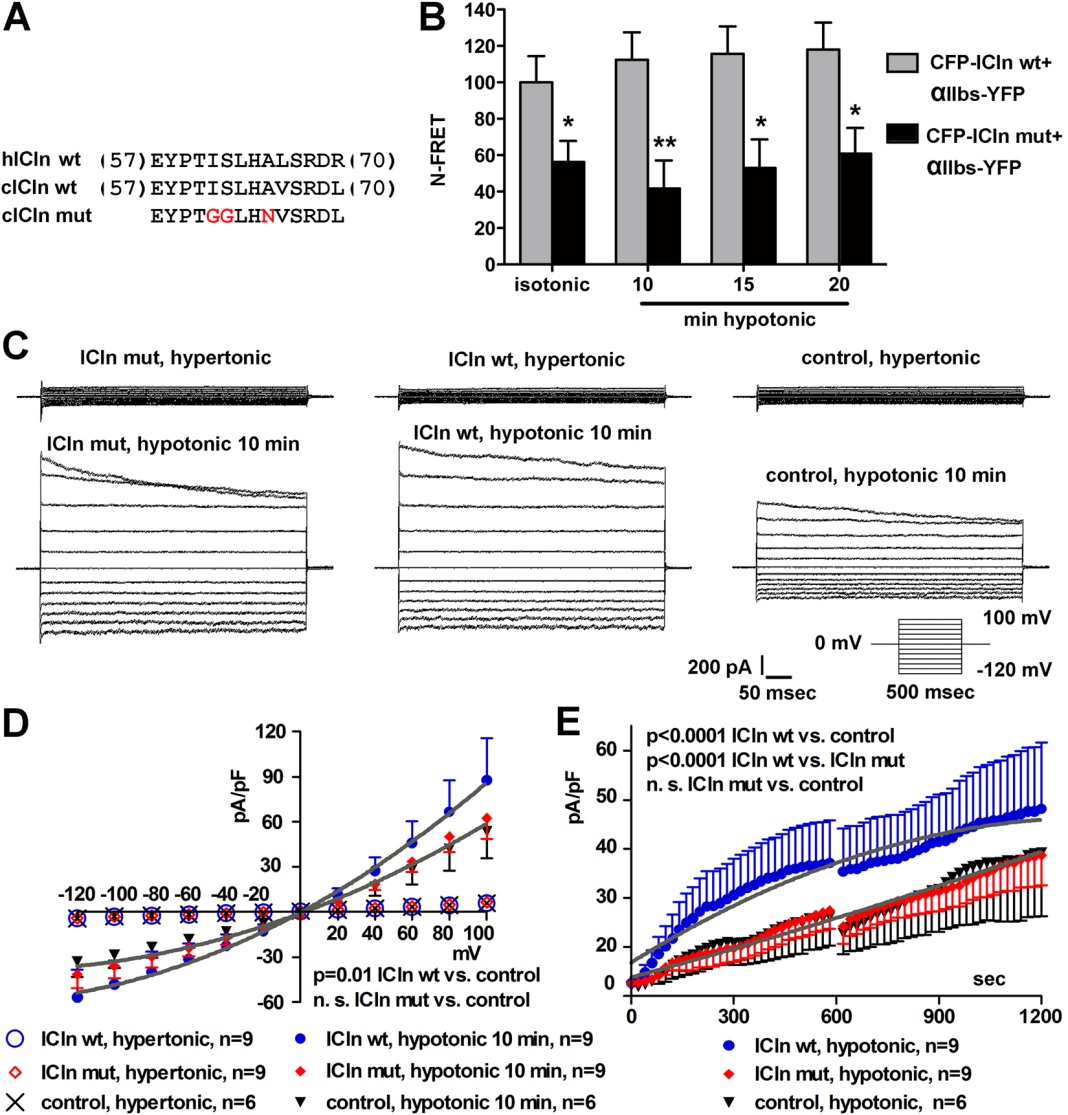
Modification of the (61)ISLHA(65) motif impairs the activation of ICl_swell_. (**A**) Sequence of the amino acids 57–70 of human (h), *Canis familiaris* (c) wild type (wt) and mutated (mut) ICln. In the latter, the amino acids establishing the strongest interaction with the integrin peptide were mutated as indicated. (**B**) Living NIH-3T3 fibroblasts were transfected with *Canis familiaris* wild type (wt) or mutated (mut) CFP-ICln and αIIb_s_-YFP. N-FRET of CFP-ICln wt and αIIb_s_-YFP (n = 13) or CFP-ICln mut and αIIb_s_-YFP (n = 9) was determined by sensitized emission in isotonic solution or after 10, 15 and 20 min of hypotonic stress. Data are normalized for the values of N-FRET of CFP-ICln wt and αIIb_s_-YFP in isotonic solution and expressed in %. ICln wt *versus* ICln mut: *p < 0.05, **p < 0.01, unpaired Student’s t-test. n refers to the number of cells. (**C**) Original recordings obtained in hypertonic (top) or hypotonic (bottom) conditions in HEK293 Phoenix cells expressing *Canis familiaris* ICln mut or wt or in control cells. Current was elicited with voltage increments of 20 mV from −120 to +100 mV applied from a holding potential of 0 mV (lower right inset). Cells overexpressed both ICln and the transfection marker EGFP as separate proteins, or EGFP only as the negative control. Experiments were done in whole-cell configuration. (**D**) Current density-to-voltage relationship measured 10 min following hypotonic shock, and (**E**) current density-to-time relationship showing upregulation of ICl_swell_ with respect to the control in ICln wt -transfected (p < 0.0001) and not in ICln mut -transfected cells. Data were fitted with second order polynomials, following application of the extra-sum of squares F test.

To further confirm the identity of the ICln amino acid(s) involved in the interaction with the integrin α chain, amino acid substitutions (I61G, S62G, A65N) were made within the integrin-recognition domain of *Canis familiaris* ICln (cICln, Fig. 6A) and FRET was determined by sensitized emission in NIH-3T3 fibroblasts transiently transfected with wild type (wt) or mutated (mut) CFP-ICln and αIIb_s_-YFP. The FRET between CFP-ICln mut and αIIb_s_-YFP measured in the plasma membrane region in isotonic and hypotonic solutions was significantly lower with respect to that measured between CFP-ICln wt and αIIb_s_-YFP (Fig. 6B). These results confirm that the ICln amino acids I61, S62 and A65 are critically involved in the interaction with αIIb_s_. To investigate the functional consequences of the impaired ICln/integrin interaction, ICl_swell_ was measured by patch-clamp experiments in whole-cell configuration in HEK293 Phoenix cells transfected with ICln wt, ICln mut or a control vector. Similar to previous results^23^, overexpression of ICln significantly up-regulated ICl_swell_. In contrast, in cells expressing ICln mut, ICl_swell_ did not differ from the endogenous current measured in control cells (Fig. 6D,E). ICln mut resulted functionally impaired compared to the wild type also following expression in NIH-3T3 fibroblasts (Supplementary Fig. S2). These results, together with those shown in Figs 2, 4 and 5, strongly indicate that interfering with the ICln/integrin interaction impairs the activation of ICl_swell_.

## Discussion

Although integrins are in general recognized as osmosensors in RVD^7,42^, the molecular mechanisms by which they influence the swelling-activated chloride current ICl_swell_ are incompletely understood. This lack of information was due in part to the molecular identity of VRACs remaining elusive for more than 30 years^43,44^. In 2014, two independent laboratories identified the leucine-rich repeat containing protein 8 A (LRRC8A) as an essential component of the channel that mediates ICl_swell_^45,46^. Recently, the structure of the human and *Mus musculus* homo-hexameric LRRC8A channel was described by cryo-electron microscopy^47–50^. Overall, these studies provide the basis for understanding the precise mechanism of activation of VRACs and identifying their molecular partners. LRRC8A and other members of the LRRC8 family form heteromers, of which the composition controls the VRAC single-channel conductance, rectification, anion selectivity and inactivation kinetics^46,51^. When reconstituted into lipid bilayers in a two-compartments system, purified LRRC8 complexes formed anion channels activated either by osmolality gradients between two compartments both containing hyperosmolar solutions, or low ionic strength in the absence of osmotic gradients between the two compartments^51^. These findings suggest that LRRC8 complexes may include a sensor for osmolarity and low ionic strength. However, other authors recently showed that low ionic strength is neither sufficient to activate VRAC on intracellular compartments, nor indispensable to keep plasma membrane-localized VRACs active, and rather suggested that the diacylglycerol/protein kinase D pathway specifically and potently activates VRAC^52^. Therefore, in a cellular context, other factors, including binding partners, post-translational modifications^53^, second messengers or even other channels^54^, may contribute to VRAC activation besides a mere reduction in the intracellular ionic strength.

The composition of the extracellular matrix modulates the characteristics of ICl_swell_ through integrin receptors, and knock-down of β1 integrin impaired ICl_swell_ activation in skeletal muscle-derived C2C12 cells^55^. The integrin β chains seem to play a fundamental role in this context. The research group of Browe and Baumgarten showed that mechanical stretch of β1 integrin leads to the activation of a chloride current with the biophysical and pharmacological fingerprints of ICl_swell_^56–60^, thus suggesting that the role of integrins in RVD would be an outside-in transduction of a mechanical stimulus with ICl_swell_ as the final effector. Accordingly, knockdown of integrin β1 also reduced RVD of adherent Ehrlich ascites cells^42^, and knockdown of integrin β3 almost completely blocked ICl_swell_ activation in basilar artery smooth muscle cells^61^. As integrin β3 co-precipitated with Src and the ClC-3 protein, these authors suggested that the integrin β3/Src/ClC-3 signalling pathway might be involved in the activation of ICl_swell_^61^.

In order to elucidate the molecular events underlying ICl_swell_ activation, we set out to explore the role of the interaction between integrins and ICln. ICln interacts with the (1020)KVGFFKR(1026) motif of the platelet-specific integrin αIIb, thereby influencing platelet activation^22,62–64^. We found that ICln can interact with the α-chain also in cells unrelated to platelets, in isotonic as well as hypotonic conditions (Fig. 1A–D). Moreover, overexpression of the transmembrane and intracellular portion of the αIIb chain (αIIb_s_) upregulated ICl_swell_ (Fig. 1E–G). Omission of the extracellular portion of the integrin αIIb chain was adopted to avoid modulation of ICl_swell_ by a possible alteration of cell adhesion to the extracellular substrate, thereby allowing for an unbiased exploration of the intracellular signalling events towards ICl_swell_ activation. Importantly, this approach showed that the ICln/integrin interaction occurs regardless of the activation state or occupancy of the integrin α-chain by extracellular ligands. These findings are in agreement with those of Larkin *et al*., which showed that ICln bound equally to purified αIIb and to integrin from resting or activated platelets, and that binding was not affected by direct integrin activation with Mn^++^ or by inhibitors of integrin occupancy^63^.

The primary sequence of the site of the integrin αIIb-chain responsible for binding to ICln was previously determined to be represented by the highly conserved (1020)KVGFFKR(1026) amino acid motif^22^, and was confirmed in the present study (Fig. 2). However, the identity of the ICln site involved in binding to the integrin α-chain remained to be established, and was determined here by NMR spectroscopy to be the (61)ISLHA(65) motif (Fig. 3). FRET experiments in NIH-3T3 cells show that cell-permeable synthetic peptides mimicking the ICln binding site to integrin or the integrin binding site to ICln not only impeded the expected hypotonicity-induced FRET increase between ICln and the plasma membrane probe YFP-mem, but also reduced FRET below the basal levels measured in isotonic conditions (Figs 2B and 4A,B). These data indicate that the cell-permeable synthetic peptides prevented the hypotonicity-induced translocation of ICln towards the cell membrane and also reduced the membrane-resident pool of ICln. To explain our findings, we propose a model (Supplementary Fig. S3) where the cytosolic, integrin-bound and membrane bound forms of ICln exist in a dynamic equilibrium. In isotonic conditions (Supplementary Fig. S3A), the cytosolic form of ICln largely prevails and a significant fraction of ICln is bound to integrin α, as denoted by FRET experiments (Fig. 1B,D). Hypotonicity (Supplementary Fig. S3B) induces the transposition of ICln towards the cell membrane (Fig. 2A), thereby altering the dynamic equilibrium in favor of the membrane-bound ICln, without a substantial increase of the integrin-bound ICln pool (Fig. 1D). The palmitoylated (1020)KVGFFKR(1026) integrin peptide may compete with the integrin α chain endogenously expressed in these cells for binding to ICln (Supplementary Fig. S3C). Such a competition would have interfered with and weakened or prevented the ICln/α chain interaction. The palmitoylated (1020)KVGFFKR(1026) peptide may have realized a non-functional interaction with ICln (not sufficiently stable, or inappropriately oriented), which failed in leading to an association of ICln to the plasma membrane. This may have produced an alteration of the dynamic equilibrium between the membrane-bound and unbound ICln in favour of the unbound form, thus leading to the observed decrease in N-FRET between ICln and the membrane probe following exposure to the hypotonic medium (Fig. 2B). These findings indicate that binding to integrin is an essential prerequisite for stabilization of ICln at the plasma membrane in hypotonicity. It is also conceivable that the palmitoylated (1020)KVGFFKR(1026) peptide may have recruited the membrane-resident ICln in a complex that was not available for interaction with the membrane probe YFP-mem. A similar reduction of FRET between ICln and the membrane probe in hypotonicity could be observed with the palmytoilated ICln peptide (Fig. 4A,B). Importantly, the lack of translocation of ICln provoked by the integrin as well as the ICln peptide was accompanied by an impaired activation of ICl_swell_ (Figs 2D,E and 4C). To have an inhibitory effect, the ICln peptide needed to be targeted to the cell membrane (Fig. 5A,B), further suggesting that its molecular target is located in close proximity of the cell periphery. We may envision that only a palmitoylated ICln peptide can reach the correct localization, sufficient local concentration or take the proper orientation to bind α integrin. Therefore, an intracellular, non-palmitoylated water-soluble ICln peptide would be inactive, while a palmitoylated membrane-targeting peptide would bind to and displace ICln from α integrin (Supplementary Fig. S3D), thus preventing the association of ICln with the plasma membrane and hampering the activation of ICl_swell_.

Mutations within the (61)ISLHA(65) ICln motif (Fig. 6A) abrogated the ICln interaction with the integrin αIIb chain (Fig. 6B) and rendered ICln inactive, *i.e*. unable to increase the ICl_swell_ intensity above the endogenous current (Fig. 6C–E). Overall, these experiments indicate that the interaction between ICln and the integrin α-chain promotes the activation of ICl_swell_. It is important to highlight that the direct physical interaction between ICln and integrin αIIb *per se* does not require α integrin interaction with extracellular ligands, as it was characterized in a cell system overexpressing a truncated form of the α-chain void of the extracellular domain. We therefore suggest that the α-chain provides a scaffolding system for ICln, facilitating the translocation and/or stabilizing the ICln protein in close proximity of the cell membrane, an essential prerequisite for ICl_swell_ activation. However, we do not exclude modulation of the ICln/integrin interaction by an outside-in signalling pathway in the physiological setting. We propose a model of integrins orchestrating the regulation of cellular volume by acting as osmosensors as well as osmoeffectors within an integrin β – ICln – integrin α - ICl_swell_ pathway (Supplementary Fig. S4). The osmotic signal, sensed by the β-chain, would trigger an intracellular signalling cascade ending on ICln, thus leading to its recruitment at the sub-membrane region and binding to the α-chain. This would promote the association of ICln with the plasma membrane, its accumulation in this compartment and activation of ICl_swell_ as the final event. It is also conceivable that ICln may dissociate from integrin prior association with the plasma membrane; this hypothesis is consistent with the mild increase of FRET between ICln and integrin α following hypotonic stress (Fig. 1D), while FRET with the membrane probe is more pronounced (Fig. 2A). The mechanism by which ICln would activate ICl_swell_ once recruited at the plasma membrane deserves further investigation. It is conceivable that ICln may take part in the macromolecular complex of the ICl_swell_ channel.

The concept of an outside-in signalling mediated by integrin and triggered by hypotonicity is well supported by several findings. In rat liver, the active conformation of integrin β1 rapidly appeared after hypo-osmotic perfusion, indicating integrin activation in response to hepatocyte swelling. Hypo-osmotic hepatocyte swelling triggered the activation of Src-type kinases, mitogen‐activated protein (MAP)-kinases and downstream metabolic events, which were fully abolished by integrin-inhibitory proteins exhibiting an Alanine-Glycine-Aspartic Acid (RGD) motif^7^. In rabbit ventricular myocytes, the activation of ICl_swell_ induced by mechanical stretch of β1 integrin was associated to an activation of focal adhesion kinases (FAK), Src, the angiotensin II receptor, and the EGF receptor^57–60^. In basilar artery smooth muscle cells, hypotonic solution increased Src phosphorylation in a time-dependent manner, a phenomenon that was significantly enhanced by integrin β3 overexpression and reduced by integrin β3 knockdown, thus suggesting that integrin β3 mediated the Src phosphorylation induced by hypotonic solution^61^.

**Figure 7:**
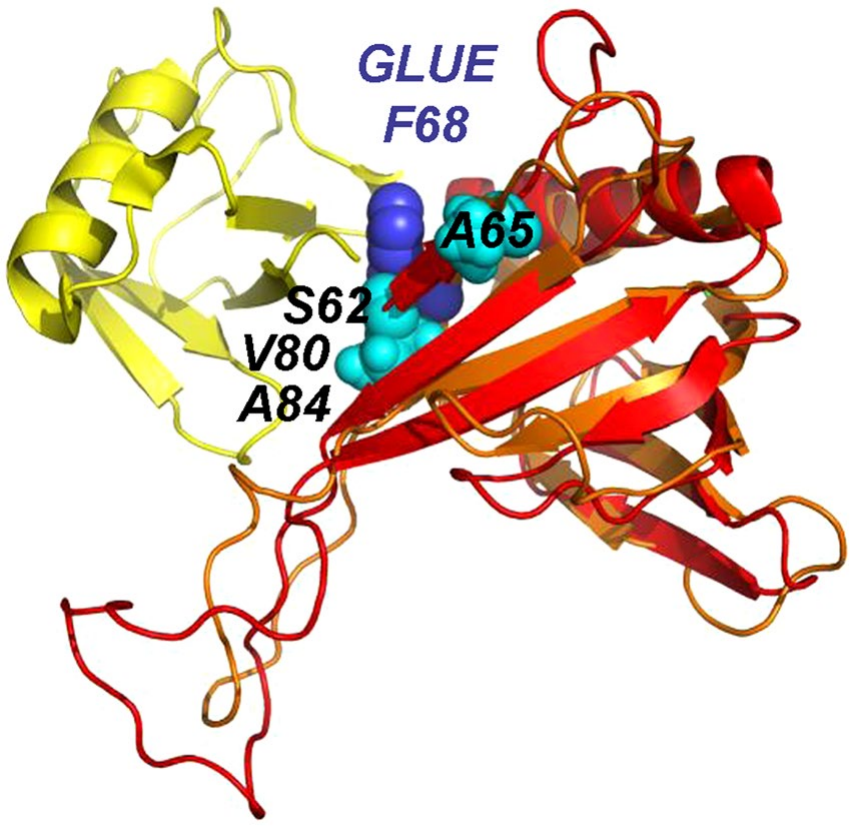
3D structural homology between ICln and the pleckstrin-homology GLUE domain of human ESCRT-II EAP45. Regions of structural similarity are indicated in red (ICln) and orange (GLUE). GLUE binding partner Ubiquitin (Ub) is shown in yellow. Most shifted residues of ICln are shown as cyan spheres. The location of GLUE residue F68 demonstrated to be crucial for the interaction with Ub is also shown in blue. Figures were prepared using the programs TopMatch^84^ and PyMOL (The PyMOL Molecular Graphics System, V. r. p., Schrödinger, LLC).

Mapping of the residues displaying frequency changes upon binding of the (1020)KVGFFKR(1026) motif on the 3D structure of ICln reveals a distinct binding site on one edge of the β-sandwich (Fig. 3C). Interestingly, this binding mode is analogous to ubiquitin (Ub) binding of the pleckstrin-homology GLUE domain of the human ESCRT-II EAP45 (also called VPS36)^65^. Figure 7 shows a structural superposition of the EAP45 GLUE-Ub complex and ICln using the pleckstrin-homology domain as templates. The structural similarity is clearly visible and convincingly supports a similar protein-binding mode. Interestingly, EAP45 GLUE binding to Ub was found to be coupled to phosphoinositol binding, thus suggesting a structural dynamic link between recognition of ubiquinated cargoes and endosomal phospholipids binding in the context of multivesicular bodies sorting. The similar protein-binding mode might reflect a functional resemblance as ICln displays a dynamic and regulated incorporation in membranes depending on changing osmotic conditions.

We could detect neither endogenous ICln nor recombinant αIIb_s_ in immunoprecipitates of recombinant αIIb_s_ or endogenous ICln from HEK293 Phoenix cells. This finding is opposed from what was observed in platelets, in which the co-immunoprecipitation was indeed detected^22^, and is indicative of a dynamic interaction, or that only a small fraction of ICln is available for interaction with integrin at a given time. This may represent a characteristic of non-platelet cells and supports the hypothesis that the physiological function of this interaction differs from that found in platelets.

Integrins and the integrin interactome are emerging as novel targets in the control of inflammatory diseases and cancer^66^. Also, as ICl_swell_ is involved in numerous pathophysiological processes^67^, its pharmacological inhibition is regarded as a potential strategy in the treatment of several life-threatening conditions. For example, in the heart, blockers of Cl^−^ currents such as ICl_swell_ may have antiarrhythmic properties^68,69^. Furthermore, pharmacological inhibition of ICl_swell_ antagonizes apoptosis by preventing apoptotic volume decrease and downstream apoptotic events^70^. Hence, ICl_swell_ inhibitors could find application in the treatment of certain forms of heart failure^56^. Although not unanimously^71^, ICl_swell_ blockers have been shown to impair the motility of cells^72^, delay their proliferation rate and induce cell cycle arrest^70^, thus suggesting that ICl_swell_ suppression might be beneficial in the treatment of some forms cancer. In brain, ICl_swell_ contributes to the release of the excitatory neurotransmitters glutamate and aspartate, and is thought to promote the excitotoxic death of neuronal cells in stroke and traumatic brain injury^3^. In addition, involvement of ICl_swell_ in the pathogenesis of exercise-induced asthma was suggested^73^. ICl_swell_ is inhibited by a wide variety of classical non-specific Cl^−^ channel blockers such as 4,4′-Diisothiocyano-2,2′-stilbenedisulfonic acid (DIDS), 5-nitro-2-(3-phenylpropyl-amino) benzoic acid (NPPB) and tamoxifen. Interestingly, tamoxifen is a selective estrogen receptor modulator that is widely used in the treatment and prevention of breast cancer (https://www.cancer.gov/about-cancer/treatment/drugs/tamoxifencitrate). ICl_swell_ blockade may in part contribute to its anti-proliferative actions. 4-[(2-Butyl-6,7-dichloro-2-cyclopentyl-2,3-dihydro-1-oxo-1H-inden-5-yl)oxy]butanoic acid (DCPIB), which is considered the most selective ICl_swell_ antagonist, attenuated microglia activation and neuronal injury in a model of ischemia/reperfusion^74^. Accordingly, astrocyte-specific LRRC8A knockout mice are protected from brain damage after ischemic stroke^75^. Overall, these lines of evidence point to a potential beneficial effect of ICl_swell_ suppression in several pathological conditions.

In summary, the protein ICln directly binds the highly conserved (1020)KVGFFKR(1026) amino acids of the intracellular domain of the integrin αIIb via the (61)ISLHA(65) amino acid motif lying in the β4 - β5 loop and β5 sheet of its pleckstrin-homology-like N-terminal portion. The stability of this interaction is required for the proper activation of the current ICl_swell_ during RVD. The findings presented here show that perturbation of the binding of ICln to the integrin α-chain leads to ICl_swell_ attenuation, and suggest that the ICln/α integrin interaction interface could be exploited as a new pharmacological target to obtain specific ICl_swell_ suppression.

## Methods

### Cell culture and transient transfection

Human embryonic kidney (HEK)293 Phoenix cells^76^ were cultured in Minimum Essential Eagle Medium (Sigma-Aldrich, St. Louis, MO, USA) supplemented with 10% fetal bovine serum (Cambrex Bio Science, Milano, Italy), 2 mM L-glutamine, 100 U/ml penicillin, 100 μg/ml streptomycin and 1 mM pyruvic acid (sodium salt). Mouse NIH-3T3 fibroblasts were cultured in Dulbecco’s modified Eagle’s medium (Sigma-Aldrich) supplemented with 10% newborn bovine serum (Cambrex Bio Science), 100 U/ml penicillin and 100 μg/ml streptomycin. The cells were maintained at 37 °C, 5% CO_2_, 95% air and 100% humidity. Subcultures were routinely established every second to third day by seeding the cells into 100 mm diameter Petri dishes following trypsin/EDTA treatment.

For FRET experiments, cells were seeded on 6-well plates and grown overnight to 80% confluence. Cells were then transfected with 8–12 μg of polyethylenimine (Sigma-Aldrich) and 2–3 μg of DNA (NIH-3T3 fibroblasts) or with 3 μg of plasmid DNA by the calcium phosphate co-precipitation method (HEK293 Phoenix cells). After 8 hours, the medium was replaced. Twenty-four hours post-transfection, cells were seeded on glass coverslips. FRET experiments were done 48 hours after transfection.

For patch-clamp experiments, cells were seeded into 30 mm diameter Petri dishes, grown overnight to 50% confluence and transfected with 3 μg of plasmid DNA by the calcium phosphate co-precipitation method (HEK293 Phoenix cells) or with 8–12 μg of polyethylenimine and 2–3 μg of DNA (NIH-3T3 fibroblasts). Eight hours after transfection, cells were transferred on glass coverslips (10 mm diameter); electrophysiology measurements were performed 24–32 hours after transfection. The transfection efficacy was about 50%. For patch-clamp experiments on untransfected cells, HEK293 Phoenix cells were seeded directly on glass coverslips and measurements were performed 16–24 hours after seeding.

### Cloning procedures, plasmid constructs and site-directed mutagenesis

Starting from human or *Canis familiaris* (from MDCK cell line) cDNA, the ORF of full length ICln was amplified by PCR using standard protocols. The cDNA sequence corresponding to amino acids 987–1039 of the human integrin αIIb chain (αIIb short - αIIb_s_) was amplified by PCR from the pcDNA3.1 vector (kindly provided by Prof. N. Moran) containing the full-length cDNA of αIIb. The PCR products encoding ICln and αIIb_s_ were cloned in frame into the mammalian expression vectors pECFP-C1 and pEYFP-N1 (Clontech, Mountain View, CA, USA) respectively, to produce the fusion proteins CFP-ICln (CFP was fused to the Nt of ICln) and αIIb_s_-YFP (YFP was fused to the Ct of ICln) suitable for FRET experiments. To label the plasma membrane, the pEYFP-Mem vector (Clontech), encoding for YFP targeted to the plasma membrane by a palmitoylation sequence (YFP-mem) was used. CFP and YFP were used as the FRET donor and acceptor, respectively.

For electrophysiology experiments, ICln or αIIb_s_ ORFs were cloned into the XhoI and BamHI restriction sites of the bicistronic mammalian expression vector pIRES2-EGFP (Clontech). The use of vectors bearing an internal ribosome entry site (IRES) allows for the simultaneous expression of two individual proteins (αIIb_s_ or ICln and the enhanced green fluorescent protein (EGFP)) from a single bicistronic mRNA without the production of fusion proteins^77^. Therefore, EGFP expression occurs only if preceded by αIIb_s_ or ICln expression. Single transfected cells could be optically individuated by detecting the fluorescence emitted by EGFP (excitation maximum = 488 nm; emission maximum = 507 nm). Control experiments were conducted in cells transfected with the pIRES2-EGFP vector with no insert and therefore overexpressing only the EGFP protein.

For protein purification, the ORFs of *Canis familiaris* ICln and its truncation mutant (ICln159X, referred to as TcICln)^40^ were cloned in frame with a hexahistidine tag into the bacterial expression vector pET3-His^78^, allowing for the expression of N-terminal His_6_-tagged proteins in *Escherichia coli* strains.

Mutagenesis of the *Canis familiaris* ICln sequence was carried out using the Site-Directed Mutagenesis kit (Stratagene, La Jolla, CA, USA) with the following primers: 5′GGAATACCCCACCGGTGGCTTGCATAACGTGTCCAGGG3′, forward, and 5′CCCTGGACACGTTATGCAAGCCACCGGTGGGGTATTCC3′, reverse. All plasmid inserts were sequenced prior to use in experiments (Microsynth AG, Switzerland).

### FRET experiments

Acceptor photobleaching experiments were performed in living or fixed (for 10 min in 3% paraformaldehyde at room temperature) NIH-3T3 or HEK293 Phoenix cells as previously described^79^. Forty-eight hours post-transfection, cells were transferred to a FCS-2 chamber (Bioptechs, Inc. Butler, PA, USA) and imaged with a TCS SP2 AOBS confocal microscope (Leica Microsystems, Wetzlar, Germany), using an argon laser and a 63X (1.4 NA) oil immersion objective. During acquisition, cells were bathed in isotonic solution (in mM: NaCl 90, KCl 5, CaCl_2_ 2, MgCl_2_ 2, Glucose 5, HEPES 10, Mannitol 80, pH 7.4) or in phosphate buffered solution. CFP was excited at 458 nm and the fluorescence acquired between 465 and 505 nm; YFP was excited at 514 nm and the fluorescence acquired between 525 and 600 nm. YFP photobleaching in whole cells was performed with 4 sequential illuminations (4 frames, 1024 × 1024 resolution, 200 Hz) at 514 nm (setting the AOTF at 100% and the zoom at 8×). FRET efficiency was calculated from the ratio of the CFP fluorescence evaluated before (CFP_pre_) and after (CFP_post_) YFP photobleaching, using the formula:

1 $${{\rm{FRET}}}_{{\rm{efficiency}}}=1-{{\rm{CFP}}}_{{\rm{pre}}}/{{\rm{CFP}}}_{{\rm{post}}}$$

in three (NIH-3T3 cells) or 4–5 (HEK293 Phoenix cells) different plasma membrane regions of interest (ROIs) for each cell. ROIs were drawn within the bleaching ROI, on the pre-bleach YFP image, in areas with a clear targeting of αIIb_s_-YFP at the cell periphery. n in Fig. 1B represents the number of analyzed cells.

For sensitized emission experiments, living NIH-3T3 cells were kept in isotonic solution for 5 minutes. The donor (CFP), FRET and acceptor (YFP) images were acquired, and the isotonic solution was replaced with hypotonic solution (in mM: NaCl 90, KCl 5, CaCl_2_ 2, MgCl_2_ 2, Glucose 5, HEPES 10, pH 7.4). Donor, FRET and acceptor images were then acquired after 10, 15 and 20 minutes. The sensitized emission (F_sen_) was calculated as already described^79,80^ and according to the formula:

2 $${{\rm{F}}}_{{\rm{sen}}}=[{{\rm{M}}}_{{\rm{IA}}}-{{\rm{M}}}_{{\rm{D}}}\cdot {\rm{\beta }}-{{\rm{M}}}_{{\rm{DA}}}\cdot ({\rm{\gamma }}-{\rm{\alpha }}\cdot {\rm{\beta }})]/({\rm{1}}-{\rm{\beta }}\cdot {\rm{\delta }})$$

M indicates the measured fluorescence (subtracted of the background) in the ROIs. Donor emission with donor excitation is indicated as M_D_, acceptor emission with donor excitation (FRET channel) is indicated as M_IA_, and acceptor emission with acceptor excitation is indicated as M_DA_. The parameters α, γ, δ and β have been calculated in samples expressing only CFP or only YFP according to^80^. The N-FRET indices were calculated in each cell from the mean intensities derived from 3 plasma membrane ROIs according to the formula:

3 $${\rm{N}}-{\rm{FRET}}={{\rm{F}}}_{{\rm{sen}}}/{{\rm{F}}}_{{\rm{D}}}$$

where F_D_ is defined as M_D_ + F_sen_. In Figs 1D and 6B, n indicates the number of analyzed cells.

Visualization of CFP- and/or YFP-expressing cells and detection of FRET in the experiments shown in the Figs 2A–C, 4A,B was performed on an IX70 inverted microscope (Olympus, Tokio, Japan) equipped with a Polychrome 4 monochromator (TILL Photonics, Planegg, Deutschland) and a cooled charge-coupled device camera (TILL Imago SVGA) controlled by the TILL Vision software (versions 3.3 and 4.0). Experiments were performed by changing three separate Olympus BX cubes equipped with the appropriate filter combinations for CFP (in nm: excitation filter 436/20, beamsplitter 455 DCLP, emission filter 480/40), YFP (in nm: excitation filter 510/20, beamsplitter 530 DCLP, emission filter 560/40), and FRET measurements (in nm: excitation filter 436/20, beamsplitter 455 DCLP, emission filter 560/40) (AHF Analysentechnik, Tübingen, Germany). FRET signals were determined in areas of interest of the cell in the membrane region before, 5 and 15 minutes after hypotonic stimulation. n indicates the number of cells analyzed; for each cell, ten ROIs were taken into account^28–30^.

To obtain the N-FRET image shown in Fig. 1C, the following image processing steps were carried out. ROIs were designed corresponding with cells expressing only CFP or YFP. From these ROIs, correction factors were measured and calculated. With these factors, sensitized emission images were obtained applying the formula (2) in a pixel-by-pixel manner using the math or the Image calculator tools of ImageJ software. The sensitized emission images were ratioed to the F_D_ image to obtain the N-FRET pictures. To suppress excessive noise in dim parts of the images, the N-FRET images were smoothed with a spatial filter and a mask was created, by setting a threshold equal to the background from this image. Subsequently, this mask was applied to the original, unfiltered N-FRET image^80^. The N-FRET images obtained were then optionally smoothed.

### Patch-clamp recordings

Patch-clamp experiments were performed as previously described^29,81^. Shortly, single cells expressing EGFP were selected by fluorescence microscopy and voltage-clamped using the whole-cell patch-clamp technique. The resistance of the glass pipettes was 3–8 MΩ when filled with the pipette solution (in mM: CsCl 125, MgCl_2_ 5, EGTA 11, raffinose 50, ATP 2, HEPES 10, pH 7.2 (adjusted with CsOH)). The hypertonic bath solution was composed of (in mM): NaCl 125, CaCl_2_ 2.5, MgCl_2_ 2.5, HEPES 10, mannitol 100, pH 7.4 (adjusted with NaOH). Fast exchange of the hypertonic bath solution with a hypotonic bath solution (in mM: NaCl 125, CaCl_2_ 2.5, MgCl_2_ 2.5, HEPES 10, pH 7.4) was obtained using a perfusion system with a flow rate of 5 ml/min and a bath volume of ~300 μl. For data acquisition, an EPC-10 amplifier (HEKA Elektronik, Lambrecht, Germany) controlled by a Macintosh computer running Patch Master (HEKA Elektronik) software was used. Access resistance as well as fast and slow capacitance were monitored and compensated throughout the recordings. All current measurements were filtered at 5 kHz and digitized at 50 kHz. To monitor the activation of the swelling activated chloride current (ICl_swell_), cells were held at 0 mV and step pulses of 400 ms duration were applied from 0 mV to 40 mV every 20 s. To establish the current to voltage (IV) relationship, step pulses of 500 ms duration were applied every 5 or 10 min from −120 mV to 100 mV in 20 mV increments from a holding potential of 0 mV. For data analysis, Fit Master (HEKA Elektronik) and EXCEL (Microsoft, USA) software were used. The current values were normalized to the membrane capacitance to obtain the current density. Each data set was obtained from cells from at least 3 independent subcultures with control experiments on the same subculture.

### Protein expression and purification

cICln and TcICln were overexpressed and labelled with ^15^N as N-terminal His_6_-tagged fusion proteins in *E. coli* BL21(DE3) strain (Agilent Technologies, Santa Clara, CA, USA). For protein expression and uniform labeling with ^15^N, transformed *E. coli* BL21 (DE3) (Agilent Technologies) were first grown in LB-medium (Sigma-Aldrich) and then diluted 1:100 into 1 liter of minimal medium (48 mM Na_2_HPO4, 22 mM KH_2_PO4, 8.56 mM NaCl and 18.7 mM ^15^NHCl (Euriso-top, Germany), supplemented with 20 ml of a 20% (w/v) glucose solution (Sigma-Aldrich), 2 ml of 1 M MgSO_4_, 0.3 ml of 1 M CaCl_2_ and 100 μg/ml ampicillin) and further cultured at 37 °C in an orbital shaker. Protein expression was induced at an OD_600_ of ~0.8 by addition of isopropyl-1-thio-β-D-galactopyranoside to a final concentration of 0.5 mM. Bacterial cells were harvested 4 h after induction. The bacterial pellet obtained from a 1 liter ^15^N labelled M9 minimal medium culture was resuspended in 30 ml of lysis buffer (50 mM K_2_HPO4, pH 8.0) and lysed using a French-press. To purify over-expressed proteins, the bacterial lysate was cleared by centrifugation and loaded onto an IMAC column (HisTrap, GE Healthcare, Chicago, IL, USA) pre-equilibrated with lysis buffer. Following extensive washing with lysis buffer, bound protein was eluted with a buffer containing 50 mM K_2_HPO4, pH 8.0 by using a NaCl gradient. Fractions containing cICln or TcICln were pooled, concentrated in Amicon Ultra 10 kDa ultrafiltration units (Millipore, Burlington, MA, USA) and loaded onto a Hiload 16/60 Superdex 75 column (GE Healthcare) equilibrated in 50 mM K_2_HPO_4_, 100 mM NaCl, pH 7.2. Fractions containing cICln or TcICln were collected, pooled and concentrated. For the following NMR experiments the buffer was changed to 50 mM K_2_HPO_4_, pH 6.6.

### NMR spectroscopy

NMR experiments were performed at 25 °C using Inova 800 MHz spectrometers (Varian Inc., Palo Alto, CA, USA). ^1^H^N^-^15^N HSQC spectra were recorded with carrier frequencies for ^1^H^N^ and ^15^N at 4.73 ppm and 120.0 ppm, respectively. The sample contained 0.8 mM recombinant canine ICln, 50 mM K_2_HPO_4_, pH 6.6 in 90% H_2_O and 10% D_2_O. All spectra were processed using NMRPipe/NMRDraw^82^ and analyzed with Sparky^83^.

### Salts, chemicals and drugs

All salts and chemicals used were of “*pro analysis”* grade. Palmitoylated and non-palmitoylated synthetic peptides were synthesized and purified by HPLC by the Organic Synthesis Core (Royal College of Surgeons, Dublin 2, Ireland) or purchased from GenScript (Piscataway, NJ, USA).

### Statistical analysis and sequence alignment

All data are expressed as arithmetic means ± S.E.M. For statistical analyses, GraphPad Prism software (version 4.03 for Windows, GraphPad Software, San Diego, CA, USA) was used. Significant differences between means were tested by paired or unpaired, two-tailed, Student’s t-test as appropriate. The current density-to-time and current density-to-voltage relationships were fitted with linear regression or second order polynomials (Y = A + BX + CX^2^). For detecting significant differences between those data, the extra-sum of squares F test was applied. Statistically significant differences were assumed at p < 0.05 (*p < 0.05; **p < 0.01; ***p < 0.001). n indicates the number of cells tested, except where otherwise specified. Sequence alignment was done with Gene Runner software, version 6.5.48.

## Supplementary information

Supplementary Information

Dataset 1

## Data Availability

The datasets used and analysed during the current study are available from the corresponding author on reasonable request.
