# Author Correction: Binding of the protein ICln to α-integrin contributes to the activation of ICl_swell_ current

**DOI:** 10.1038/s41598-019-53690-5

**Published:** 2019-11-14

**Authors:** Andreas Schedlbauer, Grazia Tamma, Simona Rodighiero, Davide Antonio Civello, Margherita Tamplenizza, Karin Ledolter, Charity Nofziger, Wolfgang Patsch, Robert Konrat, Markus Paulmichl, Silvia Dossena

**Affiliations:** 1 Department of Structural and Computational Biology, Max F. Perutz Laboratories, University of Vienna, 1030 Vienna, Austria; 2 Department of Biosciences, Biotechnologies and Biopharmaceutics, University of Bari Aldo Moro, 70126 Bari, Italy; 3 Istituto Nazionale di Biostrutture e Biosistemi, 00136 Rome, Italy; 4 Department of Experimental Oncology, European Institute of Oncology, 20141 Milan, Italy; 5 Institute of Pharmacology and Toxicology, Paracelsus Medical University, 5020 Salzburg, Austria; 6 Tensive S.r.l., 20139 Milan, Italy; 7 PharmGenetix Gmbh, 5081 Niederalm, Austria; 8 Department of Personalized Medicine, Humanomed, 9020 Klagenfurt, Austria; 9 Present Address: Centro de Investigación Cooperativa en Biociencias (CIC bioGUNE), Bizkaia Science and Technology Park, 48160 Derio (Bizkaia), Spain

Correction to: *Scientific Reports* 10.1038/s41598-019-48496-4, published online 21 August 2019

In the Supplementary Information file originally published with this Article, there was an error in Supplementary Figure S3, whereby the figure contained errors in the labelling. This error has been corrected in the Supplementary Information that now accompanies the Article.

