# Supplementary material for: Binding of the protein ICln to α-integrin contributes to the activation of IClswell current
Source: Sci Rep. 2019 Aug 21;9:12195. doi: 10.1038/s41598-019-48496-4 (PMC6704128; doi:10.1038/s41598-019-48496-4)
Supplement: Supplementary file 1 — Supplementary Information [file 41598_2019_48496_MOESM1_ESM.pdf]

Binding of the protein ICln to α-integrin contributes to the activation of ICl_{swell} current

Andreas Schedlbauer[1,9], Grazia Tamma[2,3], Simona Rodighiero[4], Davide Antonio Civello[5], Margherita Tamplenizza[6], Karin Ledolter[1], Charity Nofziger[7], Wolfgang Patsch[5], Robert Konrat[1], Markus Paulmichl[8] and Silvia Dossena[5]

[1]Department of Structural and Computational Biology, Max F. Perutz Laboratories, University of Vienna, 1030 Vienna, Austria.

[2]Department of Biosciences, Biotechnologies and Biopharmaceutics, University of Bari Aldo Moro, 70126 Bari, Italy.

[3]Istituto Nazionale di Biostrutture e Biosistemi, 00136 Rome, Italy.

[4]Department of Experimental Oncology, European Institute of Oncology, 20141 Milan, Italy.

[5]Institute of Pharmacology and Toxicology, Paracelsus Medical University, 5020 Salzburg, Austria.

[6]Tensive S.r.l., 20139 Milan, Italy.

[7]PharmGenetix Gmbh, 5081 Niederalm, Austria.

[8]Department of Personalized Medicine, Humanomed, 9020 Klagenfurt, Austria.

[9]Present address: Centro de Investigación Cooperativa en Biociencias (CIC bioGUNE), Bizkaia Science and Technology Park, 48160 Derio (Bizkaia), Spain.

Andreas Schedlbauer, Grazia Tamma and Simona Rodighiero contributed equally. Correspondence and requests for materials should be addressed to S. D. (email: silvia.dossena@pmu.ac.at)

Supplementary Table S1. FRET efficiency between CFP-ICln and αIIb_s-YFP or CFP and αIIb_s-YFP determined in NIH-3T3 fibroblasts (Fig. 1B) and HEK293 Phoenix cells (see main text). When n refers to the number of cells, FRET efficiency values determined in regions of interest (ROIs) of a same cell were averaged. The signal-to-noise ratio was obtained by the ratio between the FRET efficiency of CFP-ICln + αIIb_s-YFP and CFP + αIIb_s-YFP.

**FRET efficiency**					
**NIH-3T3 fibroblasts**					
**Sample**	**Mean value**	**S.E.M.**	**n (number of ROIs)**	**p value**	**Signal-to-noise ratio**
**CFP-ICln + αIIb_s-YFP**	9.79	1.24	69	0.0013	2.9
**CFP + αIIb_s-YFP**	3.35	0.81	51		
**Sample**	**Mean value**	**S.E.M.**	**n (number of cells)**	**p value**	**Signal-to-noise ratio**
**CFP-ICln + αIIb_s-YFP**	9.79	1.22	23	0.0015	2.9
**CFP + αIIb_s-YFP**	3.35	0.75	17		
**HEK293 Phoenix cells**					
**Sample**	**Mean value**	**S.E.M.**	**n (number of ROIs)**	**p value**	**Signal-to-noise ratio**
**CFP-ICln + αIIb_s-YFP**	2.76	0.51	41	0.0099	2.5
**CFP + αIIb_s-YFP**	1.10	0.36	41		
**Sample**	**Mean value**	**S.E.M.**	**n (number of cells)**	**p value**	**Signal-to-noise ratio**
**CFP-ICln + αIIb_s-YFP**	2.81	0.55	10	0.017	2.7
**CFP + αIIb_s-YFP**	1.05	0.39	10		

Supplementary Figure S1

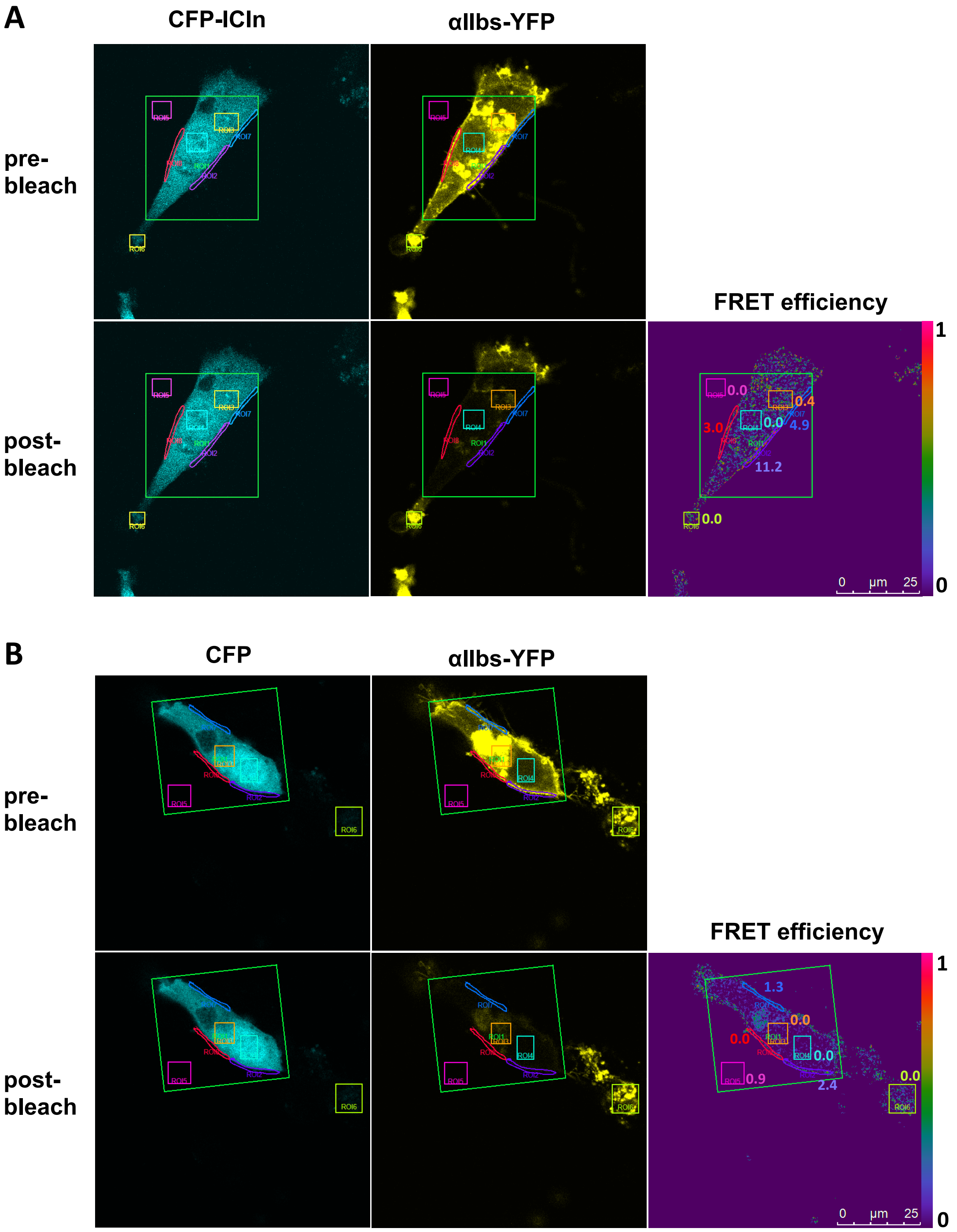

Supplementary Fig. S1. ***The integrin α-chain interacts with ICln.*** Living NIH-3T3 fibroblasts were transiently transfected with **A**, *Canis familiaris* CFP-ICln and αIIb_s-YFP or **B,** CFP and αIIb_s-YFP and bathed in isotonic solution. The FRET donor (CFP) and the FRET acceptor (YFP) were imaged

before and after photobleaching of the acceptor. The green squares correspond to the bleaching ROI and the lower right panels represent the FRET efficiency image. Numbers in color indicate the FRET efficiency (%) in a ROI of the same color. The FRET efficiency in three ROIs taken at the cell periphery based on the plasma membrane signal of αIIb_s-YFP in the pre-bleach image and also showing CFP expression was used for analysis. Control ROIs in intracellular and extracellular spaces and outside the bleaching area in two exemplary CFP-ICln and CFP-transfected cells are also shown.

Supplementary Figure S2

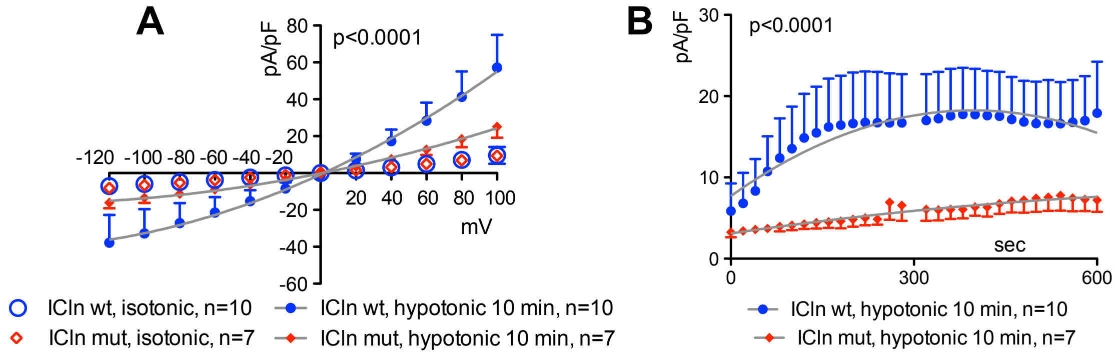

Supplementary Fig. S2. ***Modification of the (61)ISLHA(65) motif impairs the activation of ICl_{swell}.*** **A,** current density-to-voltage relationship measured 10 min following hypotonic shock, and **B**, current density-to-time relationship determined in NIH-3T3 fibroblasts transfected with *Canis familiaris* wild type (wt) or mutated (mut) ICln. Data were fitted with second order polynomials, following application of the extra-sum of squares F test.

Supplementary Figure S3

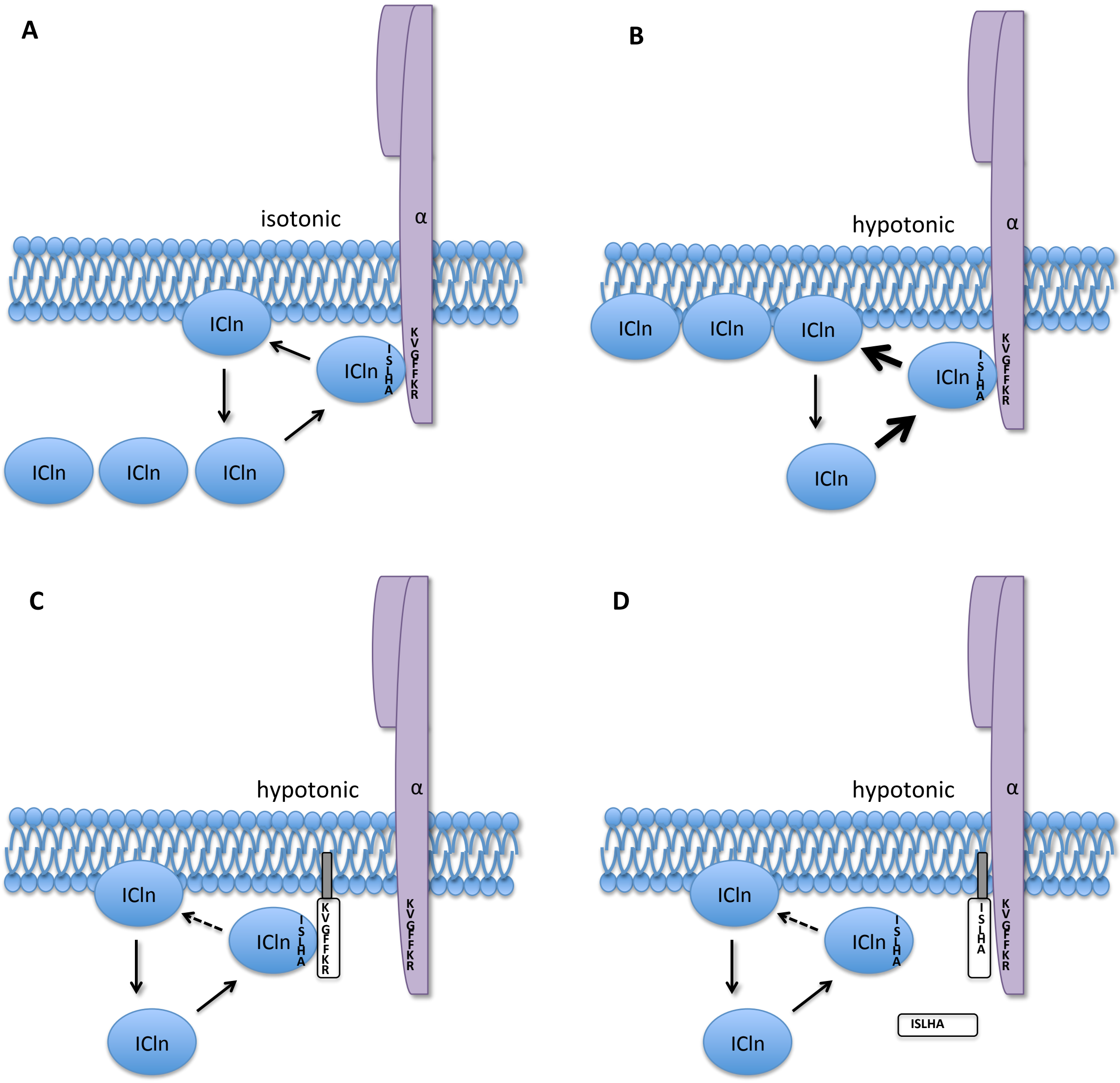

Supplementary Figure S3. Model of the dynamic equilibrium of membrane-bound, integrin bound and cytosolic ICln. A, in an isotonic environment the cytosolic form of ICln prevails. **B,** a hypotonic stimulus induces the translocation of ICln towards the cell surface and favors its accumulation at the plasma membrane. **C,** the palmitoylated membrane-targeting integrin peptide and **D,** ICln peptide, but not an unmodified, soluble ICln peptide, can interfere by competition with the ICln/integrin interaction, thus hampering the association of ICln with the plasma membrane.

Supplementary Figure S4

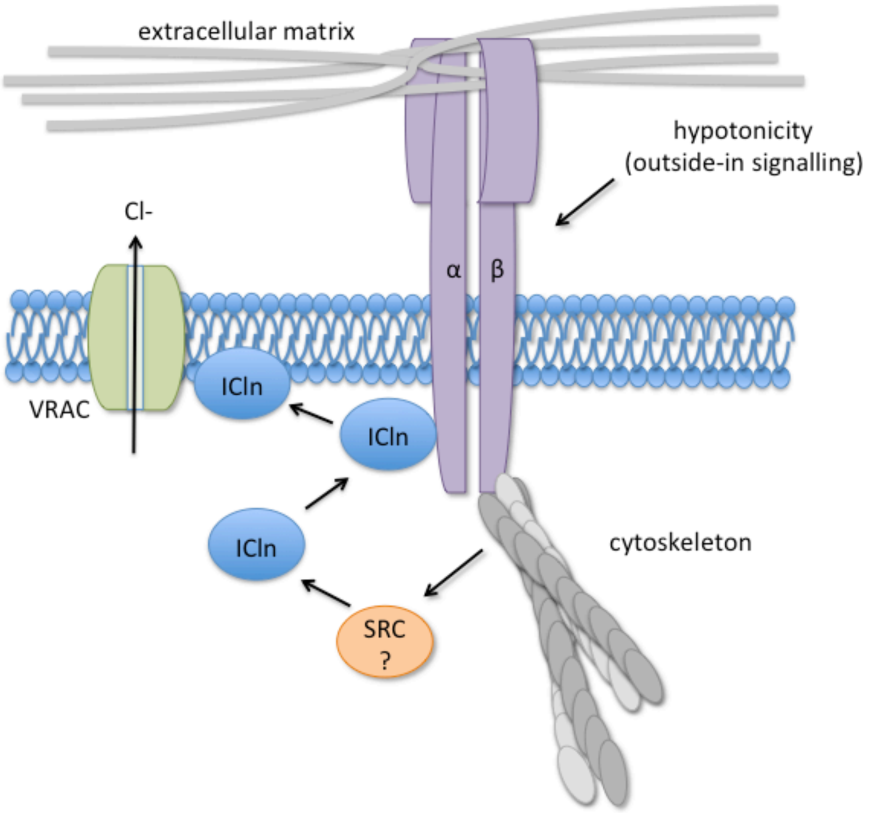

Supplementary Fig. S4. Integrin β – ICln – integrin α - ICl_{swell} pathway. An osmotic stimulus, possibly sensed by stretched integrin β, leads to activation of ICln, possibly via an unknown cytosolic player. ICln is then transposed towards the cell membrane and binds the intracellular domain of integrin α. This event enables the ICln association with the plasma membrane and activation of ICl_{swell}.
